# Survival in Idiopathic pulmonary fibrosis acute exacerbations: the non-steroid approach

**DOI:** 10.1186/s12890-015-0146-4

**Published:** 2015-12-14

**Authors:** Spyros A Papiris, Konstantinos Kagouridis, Likurgos Kolilekas, Andriana I Papaioannou, Aneza Roussou, Christina Triantafillidou, Katerina Baou, Katerina Malagari, Stylianos Argentos, Anastasia Kotanidou, Anna Karakatsani, Effrosyni D Manali

**Affiliations:** 2nd Pulmonary Medicine Department, Attikon University Hospital, Athens Medical School, National and Kapodistrian University of Athens, Athens, Greece; 7th Pulmonary Department and Asthma Center, Sotiria Chest Diseases Hospital, Athens, Greece; 6th Pulmonary Department, Sotiria Chest Diseases Hospital, Athens, Greece; 4th Pulmonary Department, Sotiria Chest Diseases Hospital, Athens, Greece; Imaging and Research Unit, Evgenidion University Hospital, National and Kapodistrian University of Athens, Athens, Greece; 1st Department of Critical Care, Evangelismos Hospital, National and Kapodistrian University of Athens, Athens, Greece

**Keywords:** Idiopathic pulmonary fibrosis acute exacerbation, Steroids, Immunosuppression, Survival

## Abstract

**Background:**

Idiopathic pulmonary fibrosis acute exacerbation (IPF-AE) constitutes IPF’s most devastating event, representing the unexpected superimposition of diffuse alveolar damage of unknown etiology. Guidelines recommend high-dose steroids treatment despite unproven benefit. We hypothesized that previous immunosuppression and the administration of high-dose steroids adversely affect IPF-AE outcome.

**Methods:**

We studied all consecutive patients hospitalized in our department for IPF deterioration from 2007 to June 2013. Our protocol consisted of immediate cessation of immunosuppression (if any), best supportive care, broad-spectrum antimicrobials and thorough evaluation to detect reversible causes of deterioration. Patients were followed-up for survival; post-discharge none received immunosuppression.

**Results:**

Twenty-four out of 85 admissions (28 %) fulfilled IPF-AE criteria. IPF-AE were analyzed both as unique events and as unique patients. As unique events 50 % survived; 3 out of 12 (25 %) in the group previously treated with immunosuppression whereas nine out of 12 (75 %) in the group not receiving immunosuppression (*p* = 0.041). As unique patients 35.3 % survived; 3 out of 6 (50 %) in the never treated group whereas three out of 11 (27.3 %) in the group receiving immunosuppression (*p* = 0.685). The history of immunosuppression significantly and adversely influenced survival (*p* = 0.035). Survival was greater in the never treated group compared to the immunosuppressed patients (*p* = 0.022). Post-discharge, our IPF-AE survivors had an 83 % 1-year survival.

**Conclusions:**

By applying the above mentioned protocol half of our patients survived. The history of immunosuppression before IPF-AE adversely influences survival. Avoiding steroids in IPF patients may favor the natural history of the disease even at the moment of its most devastating event.

## Background

Idiopathic pulmonary fibrosis (IPF) is a dreadful, chronic and irreversibly progressive disease leading to death in all patients affected and IPF acute exacerbations (IPF-AE) constitute the most devastating event during its clinical course [1, 2]. IPF-AE etiology is elusive although infections, gastroesophageal reflux disease, lung surgery, bronchoalveolar lavage, air pollution or alternatively a hypothetic acceleration of IPF fibrotic process have been suggested as triggering factors [2].

IPF-AE histological picture corresponds to the development of diffuse alveolar damage (DAD) upon usual interstitial pneumonia (UIP) [2]. This corresponds clinically to the development of an unknown etiology acute respiratory distress syndrome (ARDS) in IPF. Although IPF is an idiopathic condition with no proven therapy, the common practice for the last decades was to treat IPF with steroids and other immunosuppressants (especially azathioprine) a regimen that proved to be harmful [3–5]. Similar, by steroids, still remains the approach to treat IPF-AE as well [2–4] although there is no literature proving the effectiveness of these drugs in IPF-AE.

Convinced of its harmfulness, years before the PANTHER trial [5], the triple therapy strategy was abandoned in our everyday clinical practice and was appropriately supported in previous publications together with our adversity for the high-dose steroids regimen upon IPF-AE [1, 6]. In the present study we aimed to test the hypothesis that both a history of previous immunosuppression and the administration of high-dose steroids adversely affect IPF-AE outcome. Therefore our protocol consisted of immediate cessation of immunosuppression (if any), best supportive care, broad-spectrum antimicrobials and thorough evaluation to detect reversible causes of deterioration. All patients were followed-up for survival; after discharge none received immunosuppression.

## Methods

Firstly, we reviewed the files of all consecutive patients admitted to the pulmonary department of a tertiary teaching hospital, “Attikon” University Hospital, from January 2007 to May 2011 with the diagnosis of pulmonary fibrosis. Secondly, we isolated the cases that represented IPF according to the 2011 IPF criteria [3]. Among these admissions we further separated the ones representing IPF-AE according to the IPF Clinical Research Networks Investigators consensus (IPF diagnosis, unexplained worsening or development of dyspnea within 30 days, new lung infiltrates (mainly ground glass upon honeycomb), and exclusion of any identifiable or treatable cause of lung injury) [2]. Finally, we prospectively recorded the patients admitted with IPF and IPF-AE from June 2011 to June 2013 using the above mentioned criteria.

The protocol that we applied in every patient admitted with the diagnosis of IPF deterioration has been described in previous publications [1, 6] and consisted of immediate cessation of immunosuppression (if any), best supportive care (e.g. oxygen supplementation, painkillers, antifebrile medications, continuous monitoring of the patient etc.), broad spectrum antimicrobials according to the immune status of each patient, and thorough evaluation to detect reversible causes of respiratory deterioration. For all patients we recorded their demographics, smoking history, the last available pulmonary function tests (PFTs) and 6 min walking distance before hospitalization for AE, arterial blood gases, high-resolution computed tomography (HRCT) of the chest findings, lung biopsy, medications and comorbidities if available. Each patient underwent both HRCT and CTPA (computed tomography pulmonary angiography) to safely exclude pulmonary embolism as an etiologic factor of acute deterioration upon admission. Our patients were also evaluated with blood tests (C-reactive protein, procalcitonin, N-terminal-pro brain natriuretic peptide, troponin, complete blood count, D-dimers, erythrocyte sedimentation rate, serological studies for viruses, *Chlamydophila Pneumoniae*, *Mycoplasma Pneumoniae*), chest roentgenography, bronchoscopy, urinary antigen tests for *Streptococcus pneumoniae* and *Legionella pneumophila*, bronchoalveolar lavage (BAL), sputum sampling, nasopharyngeal aspirates, endotracheal aspiration, blood/sputum/BAL fluid cultures for microbes, mycobacteria, atypical pathogens, opportunistic infections, fungi, viruses, PFTs and cardiac echocardiography, whenever feasible taking into consideration the clinical condition of each patient. We separated admissions diagnosed as IPF-AE in 2 groups. The first group consisted of patients not receiving steroids or any other form of immunosuppressants prior to admission (treatment naïve or never treated) and the second group consisted of patients receiving prior to admission steroids or any other form of immunosuppressants (ever treated). Our treatment consisted of extended spectrum antimicrobials empirically initiated based mostly on the immunosuppression history of our patients. More precisely, patients never treated with steroids or/and immunosuppressants before the acute exacerbation event were set on antimicrobials for common gram positive, gram negative and atypical pathogens plus therapy against *influenza* virus pneumonia during the *influenza* season; patients previously treated with steroids or/and immunosuppressants received in addition to the above mentioned therapy, treatment for *Pneumocystis jirovecii* and occasionally for *cytomegalovirus* pneumonia in a documented case. All patients were followed-up after discharge for survival. None of them was treated with steroids or immunosuppression post discharge.

This work has been approved by “Attikon” hospital’s bioethics committee decision No 575/11-4-11. No patients’ consent was needed. Some of the data have been published in the form of an abstract [7].

### Statistical analysis

Categorical variables are presented as n (%). Numerical variables are presented as median (range). Comparisons between groups were performed using Cox regression analysis in order to evaluate the influence of the previous use of immunosuppressive therapy on survival. Results are presented as hazard ratios (HR) with 95 % confidence intervals (CI) in parentheses. Time to death according to previous history of immunosuppressive therapy were evaluated with Kaplan-Meier survival curves and log-rank tests. *p* values < 0.05 were considered statistically significant. Data were analyzed and graphs were created with StatsDirect 2.8.0, StatsDirect Ltd, Altrincham, Chesire, UK.

## Results

Out of 164 admissions with the diagnosis of pulmonary fibrosis from January 2007 to May 2011, 53 were attributed to IPF representing 37 unique IPF patients. From June 2011 to June 2013 another 32 cases with IPF were admitted to our department (21 unique IPF patients). In total from 2007 to 2013 we recorded 85 IPF admissions that represent 58 unique IPF patients. Diagnosis was obtained by multidisciplinary approach according to 2011 IPF guidelines [3] (Table 1). Out of these 85 IPF admissions, 24 represent IPF-AE (17 unique IPF patients) (Table 2). Fifteen IPF patients were admitted more than once, two patients presented three episodes of IPF-AE each one and one patient presented four episodes of IPF-AE.

**Table 1:** Diagnosis of IPF after multidisciplinary discussion

HRCT Pattern (*n* = 58)	Surgical Lung Biopsy Pattern (*n* = 15)	Diagnosis of IPF (*n* = 58)
UIP	UIP	7
UIP	Probable UIP	2
UIP	Possible UIP	0
UIP	no biopsy	43
Possible UIP	UIP	4
Possible UIP	Probable UIP	2

*HRCT* High Resolution Computed Tomography; *UIP* Usual interstitial pneumonia

**Table 2:** Causes of deterioration in IPF patients, *n* = 85 (100 %)

IPF exacerbation *n* = 24, (28 %)	IPF progression *n* = 12, (14 %)	Respiratory causes *n* = 38, (45 %)	Non-respiratory causes *n* = 11, (13 %)
		Respiratory infection, *n* = 35 (40 %)	Heart failure, *n* = 6 (7 %)
		*Tracheobronchitis*, *n* = *28 (33 %)*	Sepsis, *n* = 4 (5 %)
		*Pneumonia, n* = *7 (8 %)*	Pericarditis, *n* = 1 (1 %)
		Pulmonary embolism, *n* = 2 (2 %)	
		Pneumothorax, *n* = 1 (1 %)	

*IPF* idiopathic pulmonary fibrosis

Among the 85 IPF admissions an identifiable cause of the patient’s deterioration was found in 49 (58 %), that is 28 (33 %) cases of purulent tracheobronchitis, 7 (8 %) cases of pneumonia, 6 (7 %) cases of heart failure, 4 (5 %) cases of sepsis, 2 (2 %) cases of pulmonary embolism, 1 (1 %) case of pericarditis and 1 (1 %) case of pneumothorax (Table 2). Twelve patients (14 %) were diagnosed as having progression of IPF. In total, respiratory causes of deterioration other than IPF-AE were identified in 38 (45 %) of the cases. Adding the cases of IPF-AE 24 (28 %) our results showed that the most common cause of deterioration in IPF was of respiratory cause in three quarters of patients (73 %).

The descriptives of never and ever treated with steroid groups are presented in Table 3. Each one of the never and ever treated groups comprised 12 cases of IPF-AE. Ever treated patients were previously treated with steroids (10–25 mg prednisone/day) except for one patient who was on methotrexate as well due to coexisting psoriatic arthritis. The latter patient had no evidence of any other autoimmune disease that could relate to the UIP pattern observed. The present study extends chronically before the official approval of pirfenidone; as a result no study patient was on pirfenidone treatment. Total lung capacity (TLC) in the never treated group was significantly lower than in the ever treated (*p* = 0.007) whilst the need for mechanical ventilation was significantly increased in the ever treated group (*p* = 0.014). Regarding other parameters (sex, age, body mass index, smoking history, emphysema, pulmonary hypertension, other than TLC pulmonary function parameters, 6 min walking distance, level of oxygenation, radiographic pattern of IPF-AE, need for non-invasive ventilation during hospitalization, time from IPF diagnosis to the exacerbation event, previous long term oxygen therapy and BAL performed) no significant difference was found (Table 3). The majority of patients presented a normal cellular BAL pattern; that is consisting mostly of alveolar macrophages (>85 %), whereas only two patients had an abnormal BAL differential-cell pattern consisting in one case of an increase in lymphocytes and in one case in neutrophils.

**Table 3:** Descriptives of IPF-AE patients never and ever treated with steroids and immunosuppressants before hospital admission

Descriptives	IPF-AE	Never treated	Ever treated	
Sex	15 male / 9 female	6 male / 6 female	9 male / 3 female	*p* = 0.400
Age (years)	69.5 (52–82)	71.5 (52–80)	68 (52–82)	*p* = 0.270
Smoking (p-y)	40 (0–110)	35 (0–60)	42.5 (0–110)	*p* = 0.515
BMI	25.35 (17.6–32)	24.93 (22.49–31.2)	27.4 (17.6–32)	*p* = 0.081
Emphysema	4 yes, 20 no	1 yes, 11 no	3 yes, 9 no	*p* = 0.590
PH	4 yes, 6 no, 6 NA	3 yes, 6 no, 2 NA	3 yes, 4 no, 4 NA	*p* = 1
FVC (lt)	2.01 (0.59–3.17)	1.9 (0.59–2.43)	2.52 (1.34–3.17)	*p* = 0.056
FVC % predicted	63 (27.1–94)	53.15 (27.1–81.8)	65.1 (34.3–94)	*p* = 0.646
FEV_1_ (lt)	1.57 (0.56–2.78)	1.43 (0.56–1.88)	1.86 (1.25–2.78)	*p* = 0.055
FEV_1_ % predicted	60.6 (31.3–79)	53.5 (31.3–70.2)	70.2 (41.7–79)	*p* = 0.074
TLC (lt)	3.21 (2.44–4.64)	2.84 (2.44–4.23)	4.04 (2.84–4.64)	*p* = 0.007
TLC % predicted	57.2 (38.7–86.9)	52.2 (38.7–86.9)	57.5 (55.9–75)	*p* = 0.101
DLCO (ml/min/mmHg)	2.68 (1.69–4.49)	2.68 (1.69–4.49)	3.13 (1.9–3.85)	*p* = 0.619
DLCO % predicted	38.3 (19.6–54.3)	37.4 (19.6–54.3)	38.3 (24–38.9)	*p* = 0.865
6MWD (meters)	272 (150–492)	271.5 (153–492)	272 (150–441)	*p* = 0.932
Disease duration	43 (0–96)	49 (0–77)	34 (6–96)	*p* = 0.744
Pattern of GGO in HRCT	5 peripheral, 8 multifocal, 8 diffuse, 3 NA	3 peripheral, 5 multifocal, 3 diffuse, 1 NA	2 peripheral, 3 multifocal, 5 diffuse, 2 NA	*p* = 0.444
NIMV	2	0	2	*p* = 0.478
Mechanical ventilation	6	0	6	*p* = 0.014
LTOT	6	3	3	*p* = 0.640
PO_2_/fiO_2_	163.5 (66–314.29)	181.25 (116.6–266.6)	152.95 (66–314.29)	*p* = 0.378
BAL performed	6 yes, 18 no	3 yes, 9 no	3 yes, 9 no	*p* = 0.640

Data are presented as median values

*IPF-AE* idiopathic pulmonary fibrosis acute exacerbation; *BMI* body mass index; *PH* pulmonary hypertension; *FVC* (forced vital capacity); *FEV*_*1*_ forced expiratory volume in first second; *TLC* total lung capacity; *DLCO* diffusing capacity of the lung in carbon monoxide; *6MWD* six minute walking distance; *NA* not available; *GGO* ground-glass opacities; *HRCT* high resolution computerized tomography; *NIMV* non-invasive mechanical ventilation; *LTOT* long-term oxygen therapy; *PO*_*2*_ arterial pressure of oxygen; *fiO*_*2*_ fraction of inspired oxygen; *BAL* bronchoalveolar lavage

As a total out of the 24 IPF-AE cases, 12 (50 %) survived the exacerbation event. In the never treated group 9 (75 %) patients survived whereas in the ever treated group only 3 (25 %) patients did (*p* = 0.041). When examining the IPF-AE not as unique events but as unique patients (that is excluding from the analysis the subsequent IPF-AE that developed in some patients after the first exacerbation), then out of 17 IPF-AE patients, 6 (35.3 %) survived the exacerbation event. In the never treated group which comprised 6 patients, 3 (50 %) survived whereas in the ever treated group which comprised 11 patients only 3 (27.3 %) patients did (*p* = 0.685). A univariate Cox regression analysis identified the previous use of steroids as significantly and adversely influencing survival after AE in the ever and never treated groups, with a HR of 3.544 (95 % CI 1.090–11.514), *p* = 0.035 (Table 4). The same analysis in the first only exacerbation episode of the 17 unique IPF patients failed to reach statistical significance HR 1.642 (95 % CI 0.441–6.119), *p* = 0.46 and the same applies to all others variables studied (Table 4).

**Table 4:** Univariate Cox regression analysis influencing outcome in the ever and never treated IPF-AE groups

Variable	HR	95 % CI	*p*-value
Age	0.968	0.919–1.020	0.221
Gender	1.927	0.600–6.186	0.270
Smoking (pack-years)	1.000	0.980–1.020	0.996
FEV_1_ (% predicted)	1.000	0.941–1.063	0.990
FVC (% predicted)	1.001	0.961–1.043	0.943
TLC (% predicted)	1.034	0.976–1.096	0.252
DLCO (% predicted)	1.044	0.963–1.319	0.293
Charlson comorbidity index	0.729	0.466–1.143	0.169
LTOT	1.138	0.356–3.631	0.829
Pattern of GGO in HRCT	1.650	0.737–3.696	0.223
PO_2_/fiO_2_	0.990	0.980–1.001	0.067
NIMV	2.014	0.449–9.039	0.361
Mechanical ventilation	1.966	0.646–5.992	0.234
Disease duration	1.014	0.995–1.034	0.156
Previous corticosteroid use (all exacerbation events)	3.544	1.090–11.514	0.035
Previous corticosteroid use (unique patients)	1.642	0.441–6.119	0.460
BAL performed	0.676	0.188–2.427	0.548
6MWD	0.999	0.994–1.005	0.918

*IPF-AE* idiopathic pulmonary fibrosis acute exacerbation; *FVC* forced vital capacity; *FEV*_*1*_ forced expiratory volume in first second; *TLC* total lung capacity; *DLCO* diffusing capacity of the lung in carbon monoxide; *LTOT* long-term oxygen therapy; *GGO* ground glass opacities; *HRCT* high-resolution computerized tomography; *PO*_*2*_ arterial pressure of oxygen; *fiO*_*2*_ fraction of inspired oxygen; *NIMV* non-invasive mechanical ventilation; *BAL* bronchoalveolar lavage; *6MWD* six minute walking distance; *HR* hazard ratio; *CI* confidence of interval

**Fig. 1:**
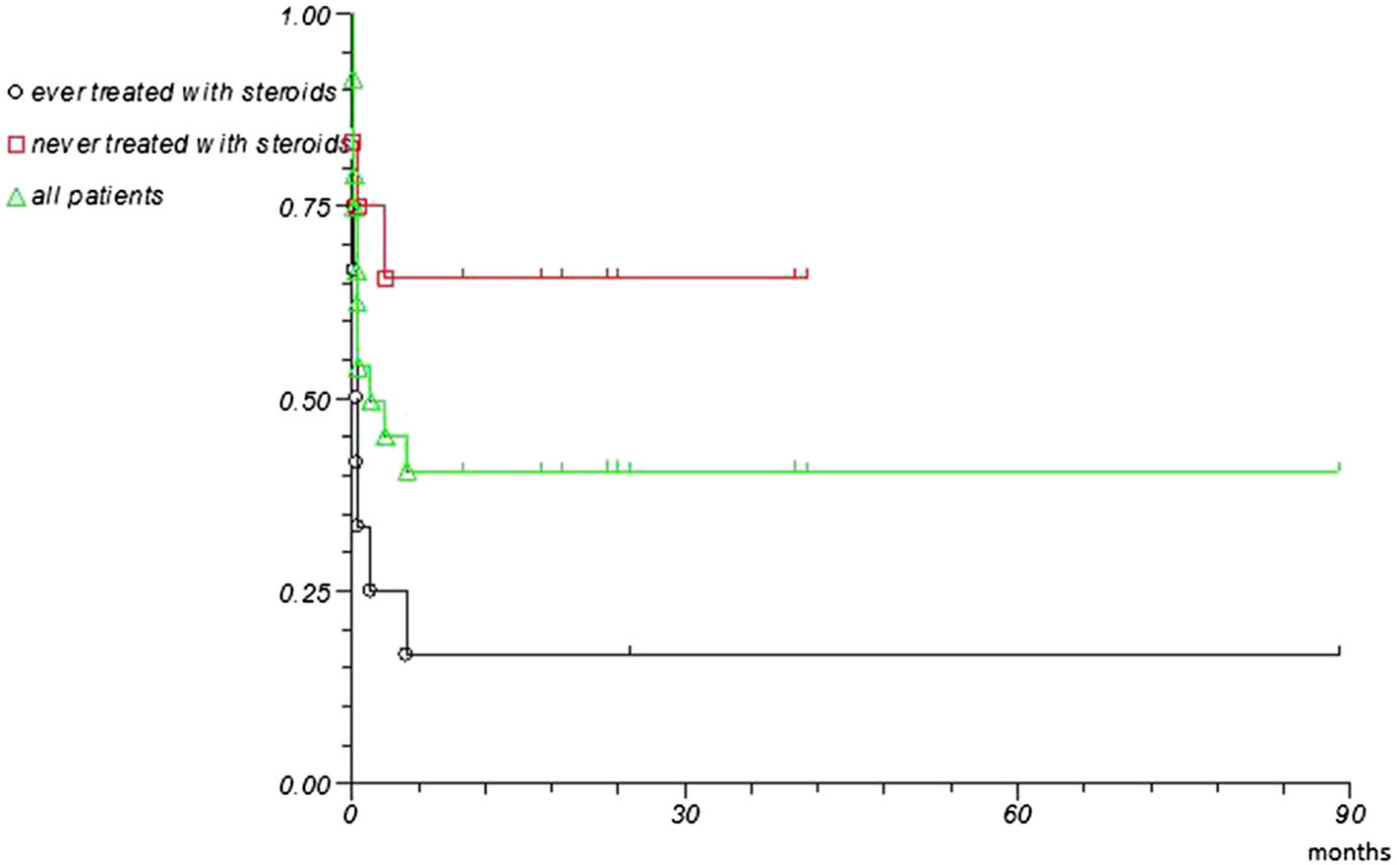
Kaplan-Meier survival curves of patients with IPF acute exacerbation ever treated (*black line*), never treated (*red line*) with steroids and immunosuppressants and of the overall population (*green line*) (*p* = 0.022)

**Fig. 2:**
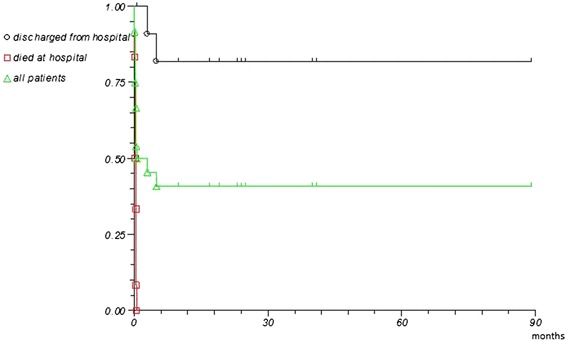
Kaplan-Meier survival curves of patients with IPF acute exacerbation treated with the study protocol after hospital discharge (*black line*) that showed an 83 % 1-year survival. Patients that died while hospitalized are shown in *red line* and the overall IPF acute exacerbation population in *green line*

Overall our IPF-AE population had a median survival of 1.73 months (range 0.1–89) with a 52 % 1-month, 45 % 3-month, 40 % 6-month and 40 % 1-year survival respectively. In the never treated group there was a 70 % 1-month, 65 % 3-month, 65 % 6-month and 65 % 1-year survival respectively (median survival could not be calculated for the never treated group since more than half of our patients were still alive at the day of census), whereas in the ever treated group the median survival was 0.43 months (range 0.2–89) with a 30 % 1-month, 22 % 3-month, 17 % 6-month and 17 % 1-year survival respectively. These differences in survival following a log-rank test were found to be statistically significant (*p* = 0.022) (Fig. 1). After discharge 83 % of survivors (i.e. patients that were discharged from the hospital after IPF-AE) of both groups were still alive on the day of census (28th February 2014). The survivors of IPF-AE treated with our protocol present a 100 % 1-month, 90 % 3-month, 83 % 6-month and 83 % 1-year survival respectively (Fig. 2).

## Discussion

This study has shown that treating rapidly deteriorating IPF patients who develop the so called IPF acute exacerbation, avoiding steroids and providing them broad spectrum antimicrobials, best supportive care and thorough evaluation to detect reversible causes of deterioration, positively influences survival since 50 % of our patients survived. Furthermore, this study has shown that patients previously treated with steroids and/or immunosuppressants presented a less favorable response to the above mentioned protocol since they showed a poorer survival in comparison to those never treated when examined either as unique events or as unique patients, although the statistical significance was reached only in the first analysis. In the case of unique patients the smaller number of patients is probably responsible for not reaching the level of statistical significance. Our findings refer to a population of ever and never treated IPF-AE patients that present similar characteristics in terms of the exacerbation’s severity. The only statistical significant differences (lower TLC in the never treated group and need for mechanical ventilation in the ever treated group) further validate our hypothesis that on the one hand provided a survival benefit in patients with lower lung capacity and on the other hand patients with higher lung capacity and comparable parameters of the exacerbation severity could not be helped by the applied protocol, thus were intubated and subsequently expired. In addition, median overall survival of those ever treated was just 0.43 months with a 17 % 1-year survival while never treated patients showed a 1-year survival of 65 %. Finally, withholding steroids in all survivors of our protocol 83 % of them were still alive 1 year after the exacerbation event.

In 2012 a randomized, double-blind, placebo-controlled trial based on an independent protocol reviewed by a committee appointed by the National Heart, Lung, and Blood Institute, and conducted in 25 clinical centers under the auspices of the Idiopathic Pulmonary Fibrosis Clinical Research Network (IPFnet) was terminated prematurely when the interim analysis demonstrated that IPF patients treated with combination therapy with prednisone, azathioprine and *N-acetylcysteine* not only had no evidence of physiological or clinical benefit but presented significantly increased rate of hospitalizations, exacerbations and deaths compared to the placebo arm [5]. Six percent of treated patients in particular developed an acute exacerbation compared with none in the placebo group. The findings of our study concerning the difference in mortality between the never and ever treated patients could further extend the results of the PANTHER trial by providing evidence that immunosuppression does not only increase the number of acute exacerbation events but also adversely affects outcome.

Based on the above evidence it appears to us inconceivable to continue managing IPF patients who precipitate in acute exacerbation being under immunosuppressive treatment with a thirty to hundred fold higher doses of steroids (from 10–35 mg prednisone for the stable IPF patient to 1000 mg methylprednisolone iv pulses for one or more days) and await improvement. The results of our study present our management approach of the rapid deteriorating patient with IPF entering exacerbation by withdrawing corticosteroids and immunosuppressants.

Our treatment, as already described, consisted of extended spectrum antimicrobials empirically initiated based mostly on the immunosuppression history of our patients. The protocol adopted by us reflects the common practice in every critical care unit where the critically ill patient developing an ARDS of unknown etiology, or during the evaluation for the detection of the etiologic factor, is managed not by high doses of pulse steroids but by extended spectrum antimicrobials since in this setting it is known that undetectable infection is the most common and the most treatable etiologic condition. Certainly by adopting the above protocol we are conscious that the results of our study are not easily comparable with previous retrospective studies describing the efficacy of specific pharmacological treatment with steroids and immunosuppressants in IPF-AE because such an alternative approach has not been examined before.

Current practice for steroids use in IPF acute exacerbations originates from the first description of IPF-AE by Kondoh and coworkers in 1993 [8] who also expressed the fundamental dilemma whether the observed DAD developing upon UIP was any etiology ARDS upon IPF or an accelerated phase of IPF itself. Kondoh and coworkers adopted the second hypothesis and treated their patients with high-dose steroids based on the common belief in those times that steroids are able to modify IPF course. Non-specifying any additional treatment and the eventual use of antimicrobials Kondoh and coworkers reported 100 % success rate for IPF AE. Both Kondoh’s hypothesis that DAD upon UIP represents an accelerated phase of IPF and Kondoh’s treatment approach with high dose steroids pulses were adopted by the international community and remain a recommendation on the current guidelines despite the fact that similar results were never reproduced by other investigators handling similar patients [3] and despite the results of the PANTHER trial [5].

In our study the never treated IPF-AE population presented a 65 % 1-year survival compared to a 1-year survival of 17 % and a median survival of only 0.43 months in the ever treated group. The overall differences in survival as discussed in the results are significant (*p* = 0.022). In contrast, the reported mortality of IPF-AE in most studies is very high, usually exceeding 50–60 % and the 3-month mortality rate following an exacerbation event reaches 60–90 % [2, 9–11]. Song and coworkers in the largest report yet published, described 90 cases of IPF-AE with a 2.2 months median survival, a 50 % in-hospital mortality and a 60 % 3-month and 73.1 % 1-year mortality [12]. In 2003 Inase and coworkers described 13 IPF-AE patients treated with methylprednisolone and 7 among them with cyclosporin A as well, that had a 1-month and 3-month mortality of 15 % and 46 % respectively; 3 patients were also treated with trimethoprim/sulfamethoxazole [13]. The same treatment (methylprednisolone, cyclosporin A, trimethoprim/sulfamethoxazole) was used by Sakamoto and coworkers to treat 11 IPF-AE patients with a mean survival of 135 days [14]. Because of the interpretation of data and the use of mean instead of median values it is quite difficult to render these results comparable to the results of other studies. However, Okamoto, in the largest study to describe the effectiveness of methylprednisolone plus cyclophosphamide or cyclosporine A in 28 patients with IPF-AE had a 1-month mortality of 85.7 % [15]. Kondoh and coworkers described 3 UIP patients (among 236 patients undergoing surgical lung biopsy for diffuse lung disease) that developed an IPF-AE and were treated with methylprednisolone pulses, immunosuppressants (2 patients) and antibiotics and presented a 33 % 3-month and 66 % 6-month mortality respectively [16]. Yokoyama described the impact of noninvasive ventilation in IPF-AE and the 11 patients presented were also treated with steroids, antibiotics and some of them with immunosuppressants as well [17]. Median survival was 30 days, and 3-month and 6-month mortality 54.5 % and 70 % respectively. Morawiec and coworkers in 2011 described 11 patients with IPF-AE and 7 with subacute exacerbation treated with steroids and cyclophosphamide that had a 100 % 1-month survival, a 72 % (55 % in the cases of IPF-AE) 3-month, 56 % 6-month and 33 % 1-year survival respectively; 6 patients received antibiotics for a suspected undocumented respiratory infection with positive outcome [18]. Finally, Simon-Blancal and coworkers treated 37 IPF-AE patients with methylprednisolone pulses and methotrexate and described a 27 % in-hospital mortality with a 4.2 months median survival, the highest ever described; 32 patients received broad spectrum antibiotics as well [19]. In many of these studies apart from methodological issues and the small number of patients it is imperative to notice that many or all of the patients received broad spectrum antibiotics. In some studies the use (or not) of antibiotics is not mentioned at all. However, to our opinion it is difficult to understand why in the majority of cases the authors do not consider the use of antibiotics as a treatment for IPF-AE and they do not consider it to be a determinant of outcome even when they use antibiotics, although experts opinion recommend the use of antibiotics in all cases of IPF-AE [20] and this reflects common practice worldwide. In our opinion the use of antibiotics is of cardinal importance to judge IPF-AE studies results and to treat IPF-AE and this is the main reason why it was included as the major therapeutic strategy in our treating protocol [6, 21].

The survival benefit in IPF-AE patients treated with broad spectrum antimicrobials instead of high dose steroids shown in the present study could be attributed to the fact that they militate the role that microbial populations play in the pathogenesis of such a condition. It is common knowledge that IPF is a disease that severely damages the lung and that IPF lung is vulnerable to any hit, even minor, that could cause DAD in the same manner that direct and indirect lung injuries cause ARDS [22]. Combining this vulnerable substrate with steroids or immunosuppressants would substantially increase the chances of microbes to induce an IPF deterioration [1, 23, 24] especially since recently it is shown that human lung microbiome and any changes in its number and composition play an important role in the pathogenesis and progression of lung diseases, IPF included [25, 26]. In the present study 40 % of our IPF patients presented deterioration due to an infectious etiology. In the literature it is shown that patients with interstitial pneumonia receiving oral steroids have 16.3 times more chances to be colonized by *Pneumocystis jirovecii* than patients not receiving steroids and 36 % of IPF patients grow bacteria in BAL fluid in the absence of clear signs of infection, even before immunosuppression [23, 27]. More recently Molyneaux and co-workers described an increased bacterial burden in BAL of IPF patients that even predicts the decline in lung function and the death of these patients [28]. Moreover a thorough review of real life patients diagnosed and treated as IPF-AE, reveals many infective and non-infective complications of steroids [29]. Wootton and coworkers applying array-based detection found evidence of viral infection in 35 % of IPF-AE [30]. All the above findings reinforce our hypothesis that IPF-AE is an ARDS considered so far of “unknown etiology” that could however be easily triggered by the most “usual suspect” named infection, a process that should be fought with priority.

It is to be noted that a limitation of the present study is the small number of patients. Furthermore, it does not include a control arm of IPF-AE patients treated with high dose steroids. The main reason for such a “limitation” was firstly our long standing clinical experience according to which IPF-AE patients treated with high dose steroids didn’t survive and secondarily to it our firm position that such a therapeutic approach would not be appropriate to treat our IPF-AE patients. Of course we are very conscious of the fact that the responsibility of such a scientific opinion that contradicts the official guidelines, although the latter are based on poor evidence, cannot be easily shouldered and that it should be incontestably proved by a prospective properly designed trial examining the role of steroids in the AE-IPF. It is common sense that a single academic center like ours could never support by itself such a trial but needs the scientific, technical and financial support of the international scientific community and of official medical institutions that could conduct a multi-center trial designed and empowered to provide definitive answers to this already long lasting and crucial for IPF patients debate. We are convinced that our proposal reflects not only our view but also the evolving view of other experts that treat IPF-AE patients worldwide [21, 31, 32].

## Conclusions

In conclusion by applying the critically ill patient protocol on the management of IPF patients developing acute exacerbation we have shown that half of our patients survived. Furthermore, we found that the history of immunosuppression before IPF-AE adversely influences survival. Avoiding steroids in IPF patients may favor the natural history of the disease even at the moment of its most devastating event.

## Abbreviations

ARDS: Acute Respiratory Distress Syndrome
BAL: Bronchoalveolar lavage
CI: Confidence of interval
DAD: Diffuse Alveolar Damage
HR: Hazard ratio
HRCT: High-resolution Computed Tomography
IPF: Idiopathic pulmonary fibrosis
IPF-AE: Idiopathic pulmonary fibrosis Acute Exacerbation
PFTs: Pulmonary Function Tests
TLC: Total lung capacity
UIP: Usual Interstitial Pneumonia
6MWD: Six minute walking distance
